# Characterization of the Lipid Metabolism in Bladder Cancer to Guide Clinical Therapy

**DOI:** 10.1155/2022/7679652

**Published:** 2022-09-12

**Authors:** Yuan-Yuan Yang, Sen-Yuan Hong, Yang Xun, Chen-Qian Liu, Jian-Xuan Sun, Jin-Zhou Xu, Meng-Yao Xu, Ye An, Deng He, Qi-Dong Xia, Shao-Gang Wang

**Affiliations:** ^1^Department and Institute of Urology, Tongji Hospital, Tongji Medical College, Huazhong University of Science and Technology, No. 1095 Jiefang Avenue, Wuhan 430030, China; ^2^Department and Institute of Gynecology and Obstetrics, Tongji Hospital, Tongji Medical College, Huazhong University of Science and Technology, No. 1095 Jiefang Avenue, Wuhan 430030, China

## Abstract

**Background:**

Bladder cancer is one of the most common malignancies of the urinary system with an unfavorable prognosis. More and more studies have suggested that lipid metabolism could influence the progression and treatment of tumors. However, there are few studies exploring the relationship between lipid metabolism and bladder cancer. This study aimed to explore the roles that lipid metabolism-related genes play in patients with bladder cancer.

**Methods:**

TCGA_BLCA cohort and GSE13507 cohort were included in this study, and transcriptional and somatic mutation profiles of 309 lipid metabolism-related genes were analyzed to discover the critical lipid metabolism-related genes in the incurrence and progression of bladder cancer. Furthermore, the TCGA_BLCA cohort was randomly divided into training set and validation set, and the GSE13507 cohort was served as an external independent validation set. We performed the LASSO regression and multivariate Cox regression in training set to develop a prognostic signature and further verified this signature in TCGA_BLCA validation set and GSE13507 external validation set. Finally, we systematically investigated the association between this signature and tumor microenvironment, drug response, and potential functions and then verified the differential expression status of signature genes in the protein level by immunohistochemistry.

**Results:**

A novel 6-lipidmetabolism-related gene signature was identified and validated, and this risk score model could predict the prognosis of patients with bladder cancer. In addition, the prognostic model was tightly related to immune cell infiltration and tumor mutation burden. Gene set variation analysis (GSVA) and gene set enrichment analysis (GSEA) showed that mTOR signaling pathway, G2M checkpoint, fatty acid metabolism, and hypoxia were enriched in patients in the high-risk score groups. Furthermore, 3 therapies specific for bladder cancer patients in different risk scores were identified.

**Conclusion:**

s. In conclusion, we investigated the lipid metabolism-related genes in bladder cancer through comprehensive bioinformatic analysis. A novel 6-gene signature associated with lipid metabolism for predicting the outcomes of patients with bladder cancer was conducted and validated. Furthermore, the risk score model could be utilized to indicate the choice of therapy in bladder cancer.

## 1. Introduction

Bladder cancer is one of the most common malignancies of the urinary system; it has the 13^th^ highest mortality among all cancers, and the mortality is still rising despite tremendous efforts that have been made for the treatment [1]. The progression of bladder cancer is a multistage process including environmental and genetic factors [2]. Previous studies indicate that tobacco smoking and occupational exposure to carcinogens are major factors [3]. The primary treatment for bladder cancer is transurethral resection of the bladder (TURB) accompanied by intravesical chemotherapy or immunotherapy [3]. However, the prognosis of patients still remains unfavorable. Therefore, it is essential to identify prognostic biomarkers to guide the selection of treatment for improving the curative effects.

Accumulating evidence demonstrates that clinical outcome, epigenetic status, and treatment resistance are associated with tumor metabolism [4, 5]. Metabolic reprogramming has been considered to be a new hallmark of malignant tumors [6]. Cancer cells usually require more energy to meet their biological needs than normal cells [7], while fatty acid oxidation is an important energy source for cancer cells, so lipid metabolism in cancer cells has been recognized as playing an important role in cancer progression [8].

Recent studies have shown that fatty acid metabolism has a close connection with bladder cancer [9]. Epidemiologic studies have shown that free fatty acid (FFA) level was increased in bladder cancer compared with paracancerous tissues. Cheng et al. demonstrated that inhibition of fatty metabolism is important in sustaining tumor growth through PPAR-*γ*-mediated pathway [10]. Besides, the molecular mechanism of drug resistance of cancer therapy might include lipid metabolism reprogramming [11]. Rysman E et al. indicated that altered lipid composition of cellular membranes could disrupt the uptake of chemotherapeutic agents and lead to chemotherapeutic resistance [12]. What is more, the tumor microenvironment (TME) plays an important role in the progression of bladder cancer, while metabolic disorders including FA metabolism changes have a crucial impact on cancer [13]. Fatty acid secreted in the microenvironment could affect the function and phenotype of infiltrating immune cells [14].

In this study, we explored the lipid metabolism-related genes in bladder cancer to conduct a model to predict patient prognosis. The prognostic risk score model independently predicted the survival outcome of bladder cancer patients. What is more, the relationship between risk score model and TME cell-infiltrating characteristics was investigated and suitable therapy treatment could be selected through the risk score model. These findings can provide a new sight into exploring the metabolic mechanism and treatment for bladder cancer.

## 2. Methods

### 2.1. Data Acquisition

We included two datasets in this study, and they are TCGA_BLCA cohort and GSE13507 cohort. The transcriptome profiles, somatic mutation profiles, and clinical information about TCGA_BLCA were downloaded from the GDC_Portal (https://portal.gdc.cancer.gov/), and the transcriptome profiles and corresponding clinical information of GSE13507 cohort were downloaded from the GEO database (https://www.ncbi.nlm.nih.gov/gds/?term=GSE13507) and served as independent external validation sets. Notably, the batch effects between TCGA_BLCA and GSE13507 cohorts were normalized by the R package “sva.”

### 2.2. Landscape of Lipid Metabolism-Related Genes in Bladder Cancer

We firstly investigated the role of lipid metabolism-related genes in TCGA_BLCA cohort, performed differential expression analysis between tumor and normal samples, and screened differentially expressed lipid metabolism-related genes with a threshold of FC (fold change, FC) > 1.5 and adjusted *p* value < 0.05. Following this, we performed univariate cox regression of these differentially expressed lipid metabolism-related genes in TCGA_BLCA cohort to further identify the critical differentially expressed lipid metabolism-related genes with significant prognostic value. Subsequently, we explored the somatic mutation of these critical genes and draw the mutation atlas.

### 2.3. Identification of the Prognostic Signature

Having systematically summarized the role of lipid metabolism-related genes in TCGA_BLCA cohort, we would like to establish a prognostic signature by these critical genes. Thus, we first randomly split all the patients in TCGA_BLCA cohort with a ratio of 1 : 1 that one for the construction and the other for the verification. Moreover, the GSE13507 cohort was served as an external independent validation cohort. Then, we separately conducted LASSO regression to screen appropriate variables and multivariate cox regression to establish the prognostic signature in the training set, and a formula of risk score based on these lipid metabolism-related genes was established:

(1) $$\begin{array}{c} \text{risk score}=\sum \text{coef}\left(i\right).\;\exp\left(i\right). \end{array}$$

*i* represents each gene in the prognostic signature, coef (i) represents the coefficient of this gene, and exp (i) is the expression value of it. Thus, each sample in training set, validation set, and GSE13507 acquired a risk score according to this formula. Moreover, we set the medium value of the risk score in training set as the threshold, and each patient received a risk level that the higher is high risk and the lower is low risk.

### 2.4. Further Verification of the Prognostic Signature

We first conducted survival analysis in all three sets to confirm the prognostic value of this signature, then performed univariate Cox regression to calculate the hazard ratio of the risk score, and carried out a meta-analysis to summarize the HR of risk score in three different sets. Besides, the differences in clinical information between high-risk patients and low-risk patients were explored by the chi-square test. Also, the ROC curves of the risk score in three sets were plotted and the area under the curves was calculated. Furthermore, we combined the indicator of tumor mutation burden and our risk score to predict the survival of patients together and explored the difference in TMB between high-/low-risk patients and the correlation between TMB and risk score. Finally, we further investigated the mutation differences between high-risk patients and low-risk patients in all TCGA_BLCA patients.

### 2.5. Gene Set Enrichment Analysis (GSEA) and Gene Set Variation Analysis (GSVA)

Having constructed and verified the prognostic signature, we wonder about the further potential mechanisms behind the risk score. Thus, we separately conducted the GSEA and GSVA in all TCGA_BLCA patients by the R package “clusterProfiler” and “GSVA.” The gene ontology (GO) gene sets, KEGG gene sets, Hallmarks gene sets, metabolism-related gene sets, and cell death-related gene sets were used to do the corresponding analysis.

### 2.6. Tumor Microenvironment, Drug Response, and Immunohistochemistry

Notably, we carried out eight different algorithms to quantify the immune cells or immune-related function of each patient according to its transcriptome profile, and they were XCELL, TIMER, QUANTISEQ, MCPCOUNTER, EPIC, CIBERSORT-ABS, CIBERSORT, and ssGSEA. Then, both differential infiltration analysis and correlation tests were conducted. Following this, we separately estimate the drug response of each patient to the commonly used drugs including cisplatin, gemcitabine, and others small molecule drugs by R package “proPhetics.” Also, the TIDE score was compared between high-risk patients and low-risk patients to predict the drug response to immunotherapy. Finally, we searched the immunohistochemistry profiles of these signature genes in the Human Protein Atlas database (HPA, https://www.proteinatlas.org/) and compared and verified the differential expression status in the protein expressed level.

### 2.7. Statistical Analysis

All the data were processed or analyzed by the R program version 4.1.1 and Microsoft Office Excel. *P* < 0.05 was regarded with significant statistical differences.

## 3. Results

### 3.1. Identification of 18 Vital Differentially Expressed Lipid Metabolism-Related Genes in Bladder Cancer

**Figure 1:**
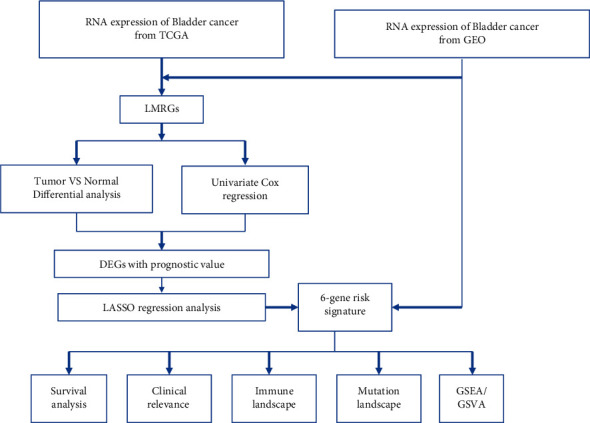
Flowchart of study design.

**Figure 2:**
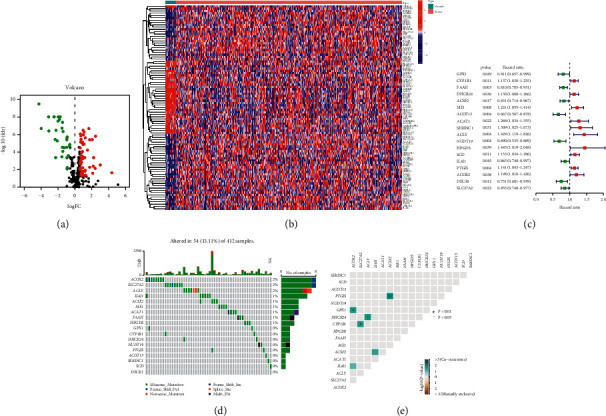
Identification of 18 vital differentially expressed lipid metabolism-related genes (LMRGs) in bladder cancer. (a) Volcano map of the expression patterns of 89 LMRGs in bladder cancer, with 57 genes upregulated and 32 genes downregulated. (b) Heat map showing the differently expressed LMRGs between normal and tumor tissues. False discovery rate (FDR) < 0.05. (c) Forrest plot of 18 LMRGs related to prognosis based on univariate analysis. (d) The mutation frequency of 18 LMRGs in 412 patients with bladder cancer from the TCGA cohort. (e) The mutation co-occurrence and exclusion analyses for 18 LMRGs.

Expression data of 309 lipid metabolism-related genes (LMRGs) were collected from the GEO and TCGA cohorts. The flow chart of this research is presented in Figure 1. 89 differently expressed LMRGs were found between normal and bladder cancer tissues, with 57 genes upregulated and 32 genes downregulated in cancer samples when the cutoff was set to FC (fold change, FC) > 1.5 and FDR<0.05 (Figures 2(a) and 2(b)). Univariate Cox regression analysis was conducted on 89 differently expressed LMRGs in TCGA_BLCA cohort. A total of 18 genes with prognostic value were identified with a *p* value < 0.05 (Figure 2(c)). The somatic mutation profile of 18 LMRGs associated with prognosis was first summarized. A total of 54 of 412 bladder cancer samples experienced mutations of LMRGs, with a frequency of 13.11%. ACOX2, SLC27A2, and ACLY had the highest mutation frequency (Figure 2(d)). Further analyses demonstrated a mutation co-occurrence relationship between ACSF2 and PTGIS, ACOX2 and GPX1, SLC27A2 and CYP1B1, and ACLY and DHCR24 (Figure 2(e)).

### 3.2. Construction and Verification of the Prognostic Index

**Figure 3:**
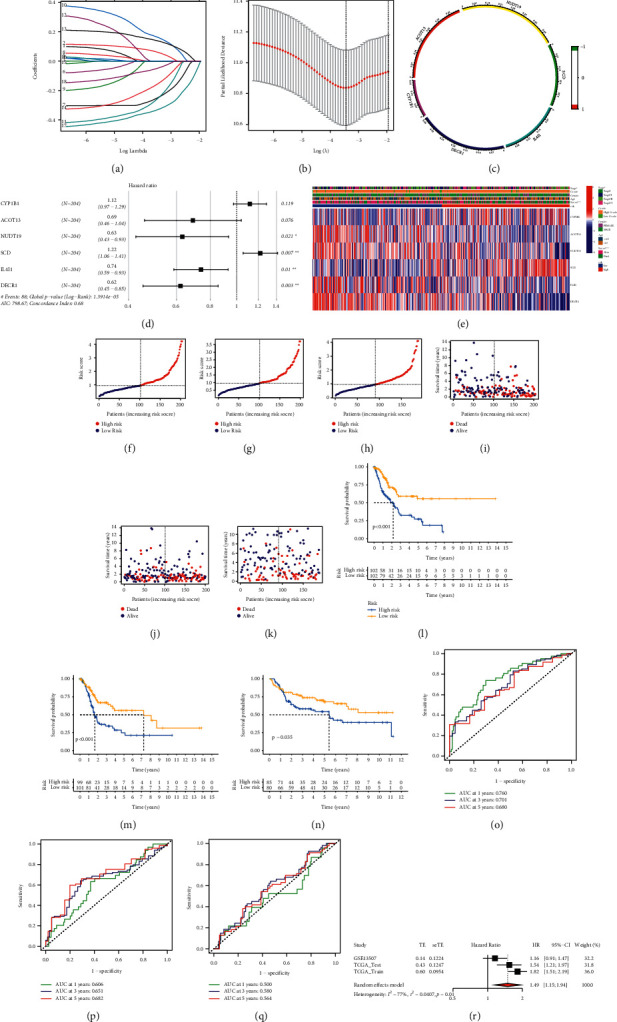
Construction and verification of the prognostic index. (a) LASSO coefficients of the 18 lipid metabolism-related genes (LMRGs). (b) Identification of genes for development of prognostic risk score model. (c) Chord diagram showed the correlation between the 6 LMRGs selected through LASSO Cox regression analysis. (d) Hazard ratio of each gene after multivariate Cox regression analysis. (e) The distribution of clinical characteristics of patients in high- and low-risk score groups. (f–k) Risk scores of patients in the training cohort (TCGA), testing cohort (TCGA), and verification cohort (GEO). (l-n) Survival status of each patient in the training cohort, testing cohort, and verification cohort. (k-m) Kaplan–Meier survival analyses of patients in the training cohort (*p* < 0.001, log-rank test), testing cohort (*p* < 0.001, log-rank test), and verification cohort (*P*=0.035, log-rank test). (o-q) AUC curve of patients in the training cohort, testing cohort, and verification cohort. (r) Meta-analysis of the training cohort, testing cohort, and verification cohort, and the results showed that the risk score was in good validity (HR = 1.49, 95% CI = 1.15–1.94, *p*=0.01).

To construct a prognostic index of lipid metabolism-related genes, we obtained 404 samples of bladder cancer from TCGA database and divided them into two groups as training cohort (*N* = 204) and testing cohort (*N* = 200). The basic characteristics of the patients included are shown in Table 1. Then, the least absolute shrinkage and selection operator (LASSO) Cox regression analysis was conducted to narrow the number of genes. Finally, six genes (CYP1B1, ACOT13, NUDT19, SCD, IL4I1, and DECR1) were used for the construction of prognostic risk score model (Figures 3(a) and 3(b)). Then, six genes were shifted from them through multivariate Cox regression (Figure 3(d)). The coefficient of these genes is displayed in Table 2. The chord diagram showed the correlation between the six genes filtered by LASSO Cox regression analysis (Figure 3(c)). To estimate the effectiveness of this model, we also collected 165 samples from GEO database (GSE13507) as validation. We then calculated the risk score of samples and divided the patients into high- and low-risk score groups according to median risk score acquired from training cohort. We also found that patients in the high-risk score group had the worse survival status than those in the low-risk group (Figures 3(f)–3(k)). We found that clinical characteristics such as stage and status were positively related to high-risk group (Figure 3(e)). The Kaplan–Meier survival analyses indicated that patients in the high-risk score group had worse survival outcome (Figures 3(l)–3(n)). The area under curve (AUC) showed the effectiveness of the prognostic index (Figures 3(o)–3(q)). Furthermore, we conducted meta-analysis based on the three cohorts to demonstrate that the risk score was in good validity (HR = 1.49, 95% CI = 1.15–1.94, *p* = 0.01) (Figure 3(r)).

### 3.3. The Relationship between Tumor Mutation Burden (TMB) and Risk Score

**Figure 4:**
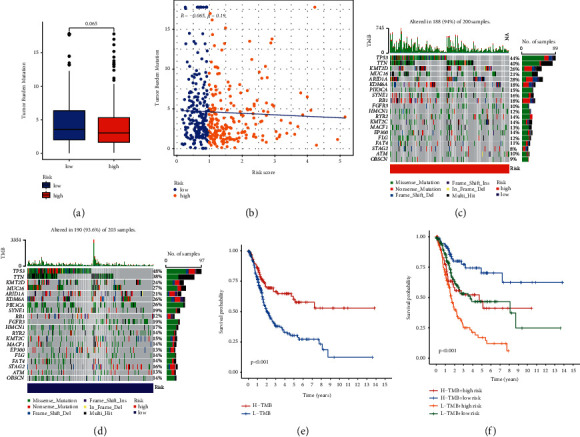
Relationship between tumor mutation burden (TMB) and risk score. (a, b) Comparison of TMB between patients in the high- and low-risk score groups, and high tumor mutation burden was associated with low-risk score. (c, d) Mutation spectrum of high-risk and low-risk patients. (e) Survival analyses for low- and high-TMB patient groups using the Kaplan–Meier curves (*P* < 0.001, log-rank test). (f) Survival analyses for four groups of TMB and risk score using the Kaplan–Meier curves. The high-TMB and low-risk score groups indicated better overall survival than the other three groups (*P* < 0.001, log-rank test).

We compared the tumor mutation burden (TMB) in high- and low-risk score patients and found the TMB of patients in the low-risk score group was higher (Figures 4(a) and 4(b)). The mutation spectrum of patients with high-risk score (Figure 4(c)) and low-risk score (Figure 4(d)) was mapped, and 14 significantly mutant genes including (ATAD5, PIK3CA, FGFR3, HUWE1, SPTAN1, GON4L, ALMS1, RELN, STAG2, AHNAK, MED13, UTRN, C2orf16, and ATR) were obtained (Table 3). Survival curves demonstrated that the prognosis of patients with high TMB was better (Figure 4(e)); furthermore, patients with high TMB and low-risk score had the best prognosis (Figure 4(f)).

### 3.4. The Relationship between Risk Score and Tumor Microenvironment (TME) in Bladder Cancer

**Figure 5:**
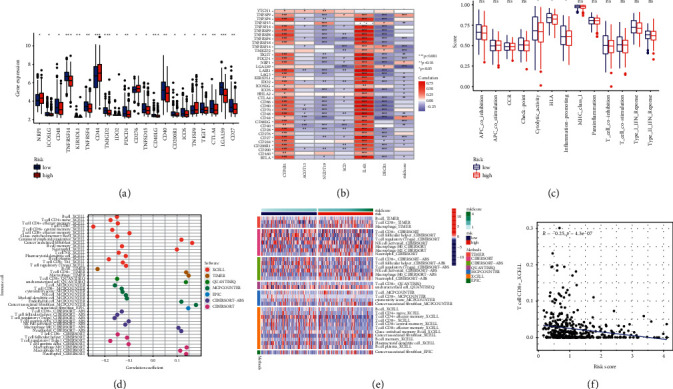
Relationship between risk score and tumor microenvironment (TME) in bladder cancer. (a) Immune checkpoint expression in high- and low-risk score patients (^*∗*^*p* < 0.05; ^*∗∗*^*p* < 0.01; ^*∗∗∗*^*p* < 0.001). (b) Correlation between immune checkpoint and six genes (^*∗*^*p* < 0.05; ^*∗∗*^*p* < 0.01; ^*∗∗∗*^*p* < 0.001). (c) Immune-related function of high- and low-risk score patients. (d) Correlation of risk score and immune cell infiltration detected by seven different methods. (e) Immune cell infiltration of patients in high- and low-risk score groups; CD8+T, Treg, and NK-activated cells were higher in low-risk score group compared with high-risk group. (f) Correlation between CD8+T-cell infiltration and risk scores.

We estimated the relationship between risk score and the immune checkpoints (ICBs), and the results indicated that NRP1, CD44, CD276, and TNFSF9 were significantly higher expressed in high-risk score patients (Figure 5(a)). Most ICBs were highly expressed in low-risk score groups (Figure 5(b)). Immune-related function analysis demonstrated that the expression of HLA, inflammation-promoting factors, and cytolytic activity was lower in high-risk score patients compared with low-risk score patients (Figure 5(c)). Then, we explored the composition of immune infiltration cells through seven methods. We found that CD8+T, Treg, and NK-activated cells were higher in the low-risk score group compared with the high-risk group (Figures 5(d) and 5(e)). In addition, CD8+T cell was negatively associated with the risk score (Figure 5(f)).

### 3.5. Gene Set Variation Analysis (GSVA) and Gene Set Enrichment Analysis (GSEA)

**Figure 6:**
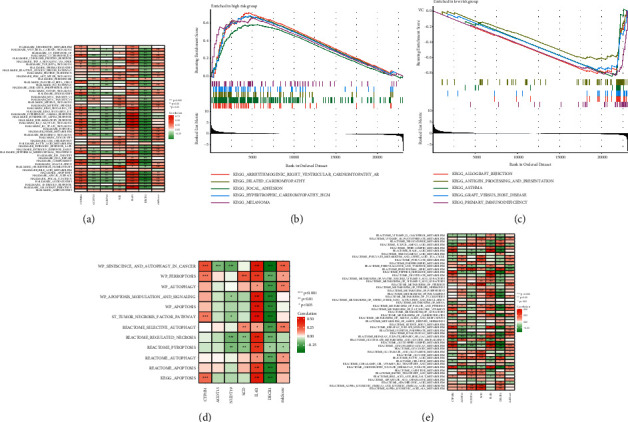
Gene set variation analysis (GSVA) and gene set enrichment analysis (GSEA). (a) GSVA in hallmark gene sets. (b, c) GSEA of patients in high- and low-risk score groups. (d) Correlation between risk score and cell death. (e) Correlation between risk score and metabolism pathways (^*∗*^*p* < 0.05; ^*∗∗*^*p* < 0.01; ^*∗∗∗*^*p* < 0.001).

We performed GSEA and GSVA to verify the correlation between risk score and pathways involved in the formation of bladder cancer. We conducted GSVA using Hallmark gene sets, and the results showed that risk score was positively associated with 31 hallmark pathways including mTOR signaling pathway, G2M checkpoint, fatty acid metabolism, and hypoxia (Figure 6(a)). We performed GSEA in different risk score groups using the Kyoto Encyclopedia of Genes and Genomes (KEGG) signaling pathways (Figures 6(b) and 6(c)). Besides, we confirmed that risk score was associated with pathways in cell death including autophagy and ferroptosis (Figure 6(d)). Also, we performed metabolism analysis and discovered risk score was positively related to fat-soluble vitamins (Figure 6(e)).

### 3.6. Response to Chemotherapy and Immunotherapy for Bladder Cancer of High- and Low-Risk Score Patients

**Figure 7:**
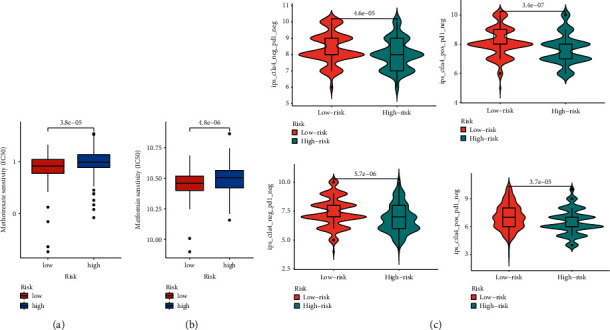
Drug response to chemotherapy or immunotherapy for bladder cancer of high- and low-risk score patients. (a) Chemotherapy drug sensitivity in high- and low-risk score patients. (b) Metformin sensitivity in high- and low-risk score patients. (c) Potential response to immunotherapy in high- and low-risk score groups. The *y*-axis means the sensitivity of these drugs, and the unit is IC50 (50% inhibiting concentration).

We estimated the response to chemotherapy drugs in different risk score groups and found that patients in the low-risk score were more sensitive to methotrexate sensitivity; furthermore, metformin could be used in bladder cancer patients of low-risk score (Figure 7(a) and 7(b)). We also conducted immunotherapy analysis between the high- and low-risk score groups and found that anti-CTLA4 and anti-PD-1 therapy could make a difference in therapeutic effect between two groups no matter whether used alone or in combination, and patients in the high-risk score groups showed better sensitivity compared with those in the low-risk score groups (Figure 7(c)).

### 3.7. Immunohistochemistry (IHC) Verification of ACOT13, CYP1, DECR1, IL4I1, and SCD

**Figure 8:**
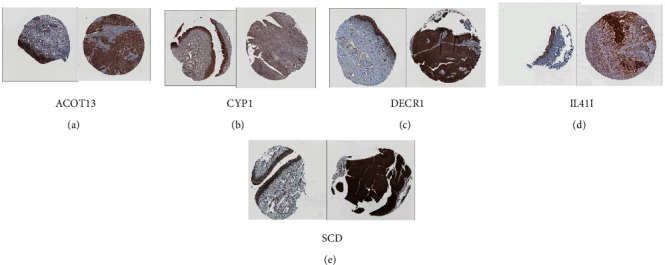
IHC staining of genes selected via LASSO Cox regression in normal tissues (left) and bladder cancer tissues (right). (a) ACOT13. (b) CYP1. (c) DECR1. (d) IL4I1. (e) SCD. The expression of CYP1 was higher in normal tissues, while other genes were higher in tumor tissues than in normal tissues.

We obtained the results of immunohistochemical staining of ACOT13, CYP1, DECR1, IL4I1, and SCD in both normal tissues and bladder cancer tissues and found that the expression of CYP1 was higher in normal tissues, while other genes were higher in tumor tissues than in normal tissues (Figure 8).

## 4. Discussion

Bladder cancer is a common malignant tumor with high rates of recurrence [15]. The treatments of bladder cancer have advanced a lot during the past decade; however, the high morbidity and mortality remain unchanged [16]. Effective therapies and prognostic markers still need to be identified.

Accumulating studies have indicated that lipid metabolism is a crucial step in metabolic reprogramming, while metabolic reprogramming is a new hallmark of malignant tumors [17]. Despite the importance of lipid metabolism in bladder cancer, few studies were conducted to explore the association between lipid metabolism and bladder cancer.

In this study, we identified the potential mechanism and prognostic value of lipid metabolism-related genes in bladder cancer via bioinformatic analysis. A 6-gene prognostic risk model was constructed by LASSO Cox analysis. In addition, these lipid metabolism-relatedgene-based signatures were tightly associated with TNM stage, T stage, N stage, and status. The patients with low-risk scores were found better outcomes than those with high-risk scores. Our findings showed that this risk model was an independent prognostic prediction for survival and was tightly associated with tumor mutation burden (TMB) and tumor microenvironment (TME).

Recently, more and more studies have suggested that the progression and prognosis of bladder cancer were tightly related to immune cell infiltration [18]. Furthermore, the functions of immune cell could be influenced by metabolic reprogramming [19]. Therefore, there must exist a relationship between risk scores and immune cell infiltration. Our results showed that patients in the high-risk score group had higher expression of M0 macrophages, while Tregs and CD8+T cells were upregulated in the low-risk group, demonstrating a differential infiltration pattern between the subgroups. In addition, patients in high-risk groups had higher expression of NRP1, CD44, CD276, and TNFSF9 than those in low-risk groups. These results indicated that the unfavorable prognosis of patients in the high-risk score groups might be due to the immunosuppressive environment and elevated expression of immune checkpoint genes [20]. We also found that tumor mutation burden was negatively associated with the risk score, and the prognosis of patient with high tumor mutation burden and low score was the best. It might be due to that tumor mutations could generate immunogenic neoantigens, thus leading to immune checkpoint blockade [21].

GSEA and GSVA of hallmarks suggested that G2M checkpoint, EMT pathway, and hypoxia were highly expressed in the high-risk score groups. MT Dillon et al. suggested that inhibition of G2M checkpoint could slow down the progression of tumors [22]. Epithelial-mesenchymal transition (EMT) is a process of epithelial cells acquiring mesenchymal features. It is associated with tumor initiation, invasion, metastasis, and resistance to therapy [23]. Hypoxia has emerged as a crucial factor in tumor pathophysiology, and microenvironment promotes altered cellular metabolism including lipid metabolism. Furthermore, reports suggested that hypoxia could trigger EMT in bladder cancer [24]. The pathways of autophagy and ferroptosis were also involved in patients of high-risk score groups, and Enyong Dai et al. suggested that autophagy-dependent ferroptosis could drive tumor-associated macrophage polarization [25]. The poorer prognosis of patients in the high-risk score groups might be tightly associated with mechanisms above.

The risk signature was constructed with ACOT13, CYP1, DECR1, IL4I1, NUDT19, and SCD ACOT13, and a member of acy1-CoA thioesterase (ACOT) enzymes can catalyze hydrolysis of fatty acyl-CoA into free fatty acids. It is usually enriched in oxidative tissues and tightly related to mitochondria [26]. Previous studies found that it was associated with many diseases, including lung cancer, pheochromocytomas, and paragangliomas [27]. CYP1 enzymes could catalyze the metabolic activation of procarcinogens and deactivation of certain anticancer drugs. Inhibition of CYP1 is an effective approach for chemoprevention, and many studies have suggested that inhibitors and prodrug target CYP1 are promising anticancer strategies [28]. DECR1 is an auxiliary enzyme of beta-oxidation, and it participates in redox homeostasis by controlling the balance between saturated and unsaturated phospholipids. Deletion of DECR1 can impair lipid metabolism and reduce tumor growth; therefore, DECR1 is important in the progression of tumor growth and treatment resistance [29]. NUDT19 has been identified to promote the proliferation, migration, and EMT process of tumor via mTORC1/P70S6K signaling pathway [30]. IL4I1 frequently associates with AHR (aryl hydrocarbon receptor) activity and activates the AHR through the generation of indole metabolites and kynurenic acid. In summary, it associates with reduced survival in patients with tumor and enhances the progression of tumor [31]. The previous study indicated that upregulation of SCD could proliferate cancer cells in a lipid-depleted environment for it could synthesize monounsaturated fatty acids. Decreased tumor SCD activity could slow tumor growth [32].

Furthermore, we identified the molecule drugs highly related to lipid metabolism genes for the treatment of bladder cancer. Methotrexate, a well-established antimetabolite, has been used separately or in combination for antitumoral activity for a while [33], and we found it suitable to treat patients with low-risk score. Excepting chemotherapy, immunotherapy is another important treatment for bladder cancer; in our study, we found that anti-CTLA4 and anti-PD1 were sensitive to patients with high-risk score of bladder cancer no matter whether used separately or in combination. These two drugs are immune checkpoint inhibition and have been licensed for the treatment of bladder cancer [34]. Metformin, often used for diabetes, is known to induce apoptosis in many types of cancers and has the feasibility as a drug repositioning used for the treatment of bladder cancer [35]. Our study indicated that patients in the low-risk score groups were suitable for the treatment of metformin compared with patients in the high-risk score groups.

Our study constructed and validated a prognostic signature model based on lipid metabolism genes, which could predict the prognosis of patients with bladder cancer well and guide the treatment for patients with bladder cancer. Our study also has limitations, and the main limitation of the study is that we do not have experimental studies in vivo and in vitro. Further studies would be conducted to validate what roles the lipid metabolism-related genes play in bladder cancer.

## 5. Conclusions

In conclusion, we investigated the lipid metabolism-related genes in bladder cancer through comprehensive bioinformatic analysis. A novel 6-gene signature associated with lipid metabolism for predicting the outcomes of patients with bladder cancer was conducted and validated. Furthermore, the risk score model could be utilized to indicate the choice of therapy in bladder cancer.

## Figures and Tables

**Table 1:** Basic characteristics of included BLCA patients.

	Overall	GSE13507	TCGA_Test	TCGA_Train	*p*
*n*	569	165	200	204	
Age (mean (SD))	67.25 (11.10)	65.18 (11.97)	67.77 (11.11)	68.42 (10.12)	0.014
Gender = female/male (%)	135/434 (23.7/76.3)	30/135 (18.2/81.8)	47/153 (23.5/76.5)	58/146 (28.4/71.6)	0.071
Grade (%)					<0.001
High grade	440 (77.3)	60 (36.4)	186 (93.0)	194 (95.1)	
Low grade	126 (22.1)	105 (63.6)	12 (6.0)	9 (4.4)	
Unknown	3 (0.5)	0 (0.0)	2 (1.0)	1 (0.5)	
T (%)					<0.001
T1	83 (14.6)	80 (48.5)	3 (1.5)	0 (0.0)	
T2	150 (26.4)	31 (18.8)	56 (28.0)	63 (30.9)	
T3	210 (36.9)	19 (11.5)	100 (50.0)	91 (44.6)	
T4	69 (12.1)	11 (6.7)	23 (11.5)	35 (17.2)	
Ta	24 (4.2)	24 (14.5)	0 (0.0)	0 (0.0)	
Unknown	33 (5.8)	0 (0.0)	18 (9.0)	15 (7.4)	
M (%)					<0.001
M0	352 (61.9)	158 (95.8)	102 (51.0)	92 (45.1)	
M1	18 (3.2)	7 (4.2)	6 (3.0)	5 (2.5)	
MX	197 (34.6)	0 (0.0)	91 (45.5)	106 (52.0)	
Unknown	2 (0.4)	0 (0.0)	1 (0.5)	1 (0.5)	
*N* (%)					<0.001
N0	386 (67.8)	151 (91.5)	116 (58.0)	119 (58.3)	
N1	54 (9.5)	8 (4.8)	21 (10.5)	25 (12.3)	
N2	79 (13.9)	4 (2.4)	37 (18.5)	38 (18.6)	
N3	8 (1.4)	1 (0.6)	3 (1.5)	4 (2.0)	
NX	37 (6.5)	1 (0.6)	21 (10.5)	15 (7.4)	
Unknown	5 (0.9)	0 (0.0)	2 (1.0)	3 (1.5)	
Status = alive/dead (%)	324/245 (56.9/43.1)	96/69 (58.2/41.8)	112/88 (56.0/44.0)	116/88 (56.9/43.1)	0.916
Risk score (median (IQR))	0.96 [0.66, 1.46]	0.97 [0.65, 1.47]	0.95 [0.66, 1.45]	0.96 [0.66, 1.45]	0.85
Risk = high/low (%)	286/283 (50.3/49.7)	85/80 (51.5/48.5)	99/101 (49.5/50.5)	102/102 (50.0/50.0)	0.925

**Table 2:** Signature genes and their coefficient.

Symbol	Coef
CYP1B1	0.11119677191537
ACOT13	−0.371344139411004
NUDT19	−0.461089925531869
SCD	0.199677207803535
IL4I1	−0.302776856487651
DECR1	−0.475644491902455

**Table 3:** Mutation atlas differences between high-risk and low-risk patients.

Gene	H-wild	H-mutation	L-wild	L-mutation	*p* value
ATAD5	195 (97.5%)	5 (2.5%)	183 (90.15%)	20 (9.85%)	0.004334171
PIK3CA	170 (85%)	30 (15%)	151 (74.38%)	52 (25.62%)	0.011636589
FGFR3	181 (90.5%)	19 (9.5%)	165 (81.28%)	38 (18.72%)	0.011988853
HUWE1	19 6 (98%)	4 (2%)	187 (92.12%)	16 (7.88%)	0.012810357
SPTAN1	188 (94%)	12 (6%)	175 (86.21%)	28 (13.79%)	0.014308358
GON4L	195 (97.5%)	5 (2.5%)	187 (92.12%)	16 (7.88%)	0.027357379
ALMS1	192 (96%)	8 (4%)	183 (90.15%)	20 (9.85%)	0.034494781
RELN	192 (96%)	8 (4%)	183 (90.15%)	20 (9.85%)	0.034494781
STAG2	183 (91.5%)	17 (8.5%)	171 (84.24%)	32 (15.76%)	0.037673236
AHNAK	189 (94.5%)	11 (5.5%)	179 (88.18%)	24 (11.82%)	0.037837752
MED13	191 (95.5%)	9 (4.5%)	182 (89.66%)	21 (10.34%)	0.040835835
UTRN	191 (95.5%)	9 (4.5%)	182 (89.66%)	21 (10.34%)	0.040835835
C2orf16	195 (97.5%)	5 (2.5%)	188 (92.61%)	15 (7.39%)	0.042331744
ATR	193 (96.5%)	7 (3.5%)	185 (91.13%)	18 (8.87%)	0.042692803

## Data Availability

The source data of this study were derived from the public repositories, as indicated in the section of “Methods” of the manuscript. All data that support the findings of this study are available from the corresponding author upon reasonable request.

## References

[1] Cumberbatch M. G. K., Jubber I., Black P. C. (2018). Epidemiology of bladder cancer: a systematic review and contemporary update of risk factors in 2018. *European Urology*.

[2] Witjes J. A., Bruins H. M., Cathomas R. (2021). European association of urology guidelines on muscle-invasive and metastatic bladder cancer: summary of the 2020 guidelines. *European Urology*.

[3] Babjuk M., Burger M., Compérat E. M. (2019). European association of urology guidelines on non-muscle-invasive bladder cancer (tat1 and carcinoma in situ) - 2019 update. *European Urology*.

[4] Karasinska J. M., Topham J. T., Kalloger S. E. (2020). Altered gene expression along the glycolysis-cholesterol synthesis axis is associated with outcome in pancreatic cancer. *Clinical Cancer Research*.

[5] Cristea S., Coles G. L., Hornburg D. (2020). The MEK5-ERK5 Kinase axis controls lipid metabolism in small-cell lung cancer. *Cancer Research*.

[6] Thakur C., Chen F. (2019). Connections between metabolism and epigenetics in cancers. *Seminars in Cancer Biology*.

[7] Beloribi-Djefaflia S., Vasseur S., Guillaumond F. (2016). Lipid metabolic reprogramming in cancer cells. *Oncogenesis*.

[8] Iwamoto H., Abe M., Yang Y. (2018). Cancer lipid metabolism confers antiangiogenic drug resistance. *Cell Metabolism*.

[9] Massari F., Ciccarese C., Santoni M. (2016). Metabolic phenotype of bladder cancer. *Cancer Treatment Reviews*.

[10] Cheng S., Wang G., Wang Y. (2019). Fatty acid oxidation inhibitor etomoxir suppresses tumor progression and induces cell cycle arrest via PPAR*γ*-mediated pathway in bladder cancer. *Clinical Science*.

[11] Cao Y. (2019). Adipocyte and lipid metabolism in cancer drug resistance. *Journal of Clinic Investigation*.

[12] Hoy A. J., Nagarajan S. R., Butler L. M. (2021). Tumour fatty acid metabolism in the context of therapy resistance and obesity. *Nature Reviews Cancer*.

[13] Elia I., Haigis M. C. (2021). Metabolites and the tumour microenvironment: from cellular mechanisms to systemic metabolism. *Nature Metabolism*.

[14] Man K., Kallies A., Vasanthakumar A. (2022). Resident and migratory adipose immune cells control systemic metabolism and thermogenesis. *Cellular Molecular Immunol*.

[15] Patel V. G., Oh W. K., Galsky M. D. (2020). Treatment of muscle-invasive and advanced bladder cancer in 2020. *CA: A Cancer Journal for Clinicians*.

[16] Kim I. H., Lee H. J. (2021). Perioperative systemic treatment for muscle-invasive bladder cancer: current evidence and future perspectives. *International Journal of Molecular Sciences*.

[17] Liu J., Peng Y., Shi L. (2021). Skp2 dictates cell cycle-dependent metabolic oscillation between glycolysis and TCA cycle. *Cell Research*.

[18] Shi S., Ma T., Xi Y. (2021). Characterization of the immune cell infiltration landscape in bladder cancer to aid immunotherapy. *Archives of Biochemistry and Biophysics*.

[19] Morrissey S. M., Zhang F., Ding C. (2021). Tumor-derived exosomes drive immunosuppressive macrophages in a pre-metastatic niche through glycolytic dominant metabolic reprogramming. *Cell Metabolism*.

[20] Chen X., Xu R., He D. (2021). CD8^+^ T effector and immune checkpoint signatures predict prognosis and responsiveness to immunotherapy in bladder cancer. *Oncogene*.

[21] Pei J., Li Y., Su T. (2020). Identification and validation of an immunological expression-based prognostic signature in breast cancer. *Frontiers in Genetics*.

[22] Dillon M. T., Good J. S., Harrington K. J. (2014). Selective targeting of the G2/M cell cycle checkpoint to improve the therapeutic index of radiotherapy. *Clinical Oncology*.

[23] Pastushenko I., Blanpain C. (2019). EMT transition states during tumor progression and metastasis. *Trends in Cell Biology*.

[24] Joseph J. P., Harishankar M. K., Pillai A. A., Devi A. (2018). Hypoxia induced EMT: a review on the mechanism of tumor progression and metastasis in OSCC. *Oral Oncology*.

[25] Dai E., Han L., Liu J. (2020). Autophagy-dependent ferroptosis drives tumor-associated macrophage polarization via release and uptake of oncogenic KRAS protein. *Autophagy*.

[26] Du N., Dong D., Sun L. (2021). Identification of ACOT13 and PTGER2 as novel candidate genes of autosomal dominant polycystic kidney disease through whole exome sequencing. *European Journal of Medical Research*.

[27] Dahia P. L. M. (2013). Novel hereditary forms of pheochromocytomas and paragangliomas. *Front Horm Res*.

[28] Cui J., Li S. (2014). Inhibitors and prodrugs targeting CYP1: a novel approach in cancer prevention and therapy. *Current Medicinal Chemistry*.

[29] Blomme A., Ford C. A., Mui E. (2020). 2, 4-dienoyl-CoA reductase regulates lipid homeostasis in treatment-resistant prostate cancer. *Nature Communication*.

[30] Lan C., Wang Y., Su X., Lu J., Ma S. (2021). LncRNA LINC00958 Activates mTORC1/P70S6K signalling pathway to promote epithelial-mesenchymal transition process in the hepatocellular carcinoma. *Cancer Investigation*.

[31] Sadik A., Somarribas Patterson L. F., Öztürk S. (2020). IL4I1 is a metabolic immune checkpoint that activates the AHR and promotes tumor progression. *Cell*.

[32] Lien E. C., Westermark A. M., Zhang Y. (2021). Low glycaemic diets alter lipid metabolism to influence tumour growth. *Nature*.

[33] Kern S., Truebenbach I., Höhn M. (2019). Combined antitumoral effects of pretubulysin and methotrexate. *Pharmacol Res Perspect*.

[34] Dyck L., Mills K. H. G. (2017). Immune checkpoints and their inhibition in cancer and infectious diseases. *European Journal of Immunology*.

[35] Jang J. H., Sung E. G., Song I. H., Lee T. J., Kim J. Y. (2020). Metformin induces caspase-dependent and caspase-independent apoptosis in human bladder cancer T24 cells. *Anticancer Drugs*.

